# The Effects of Dietary Herbal Powders on Growth, Intestinal Physiology and Meat Quality of Quails (*Coturnix japonica*)

**DOI:** 10.3390/vetsci13070723

**Published:** 2026-07-22

**Authors:** İsa Coşkun, Hüseyin Çayan, Hayrettin Çayıroğlu, Mehmet Akif Boz, Emine Doğan, Emine Bilginoğlu

**Affiliations:** 1 Department of Animal Science, Faculty of Agriculture, Kırşehir Ahi Evran University, Kırşehir 40100, Türkiye; huseyin.cayan@ahievran.edu.tr (H.Ç.); hayrettincayiroglu@ahievran.edu.tr (H.Ç.); 2 Department of Animal Science, Faculty of Agriculture, Yozgat Bozok University, Yozgat 66900, Türkiye; m.akif.boz@bozok.edu.tr; 3 Laboratory Technician and Veterinary Health Program, Çiçekdağı Vocational School, Kırşehir Ahi Evran University, Kırşehir 40700, Türkiye; emine.dogan@ahievran.edu.tr; 4 Department of Field Crops, Faculty of Agriculture, Kırşehir Evran University, Kırşehir 40100, Türkiye; emine.bilginoglu@ahievran.edu.tr

**Keywords:** quails, herbal powders, nutrition, growth, gut health

## Abstract

The aim of this study was to determine the effects of supplementing quail diets with 0.5% thyme, sage, mint, and oregano powder on nutritional performance. A total of 400 one-day-old quail chicks, standardized for live weight, were randomly assigned to five treatment groups with four replicates per group (20 chicks per replicate). The groups were allocated to treatments as *C*—control, *T*—thyme, *S*—sage, *M*—mint, and *O*—oregano. The experiment lasted for 35 days. Chicks were reared in cages sized 70 × 90 × 60 cm, with a 10 cm layer of wood shavings on the ground. Feed and water were provided ad libitum. At the end of the experiment, live weight gain and lactic acid bacteria (LAB) colonization in ceca were significantly higher in the *T* (thyme) and *O* (oregano) groups compared to the *C*, *S* and *M* groups (*p* < 0.05). Malondialdehyde (MDA) levels in the breast meat were lower in the *S*, *M* and *O* groups compared to the *C* group. Jejunal villi length was significantly greater in the *S* and *O* groups compared to the others (*p* < 0.05). In the ileum, villi height was higher in the *T* group than in the other groups (*p* < 0.05). The villi height/crypt depth ratio in the jejunum was significantly higher in the *T* and *O* groups compared to the *C* group (*p* < 0.05), whereas in the ileum, this ratio was lower in the *S* group than in the other groups. Mucin-2 level in the ileum was significantly reduced in the *S*, *M* and *O* groups compared to the *C* group (*p* < 0.05) and was similar between *T* and *C*. In conclusion, dietary supplementation with vegetative powder of thyme and oregano can be used to improve growth, intestinal villi morphology and LAB colonization in quails. Also, low-dose herbal powder supplementation can be used to suppress MDA formation in the breast meat stored at 4 °C for 3 days.

## Simple Summary

Many aromatic plants belonging to the Lamiaceae family (such as thyme, oregano, sage, and mint) have been used in numerous studies at various doses to improve performance, gut health, and meat quality of poultry. These products are expensive and difficult to obtain and, therefore, they are intended for use as feed additives, and they must be included in the diets at lower doses. This study was conducted to find which of these plants—thyme, oregano, sage, and mint—added at a low dose (0.5%) to quail diets was more effective in terms of performance, gut health, and meat quality characteristics. While the addition of low doses of thyme and oregano improved performance, the addition of all aromatic herbs increased villi development in the intestine and suppressed MDA formation in breast meat and suppressed the mucin 2 gene in the ileum, except for the supplementation of thyme. The additions of thyme, mint, and oregano increased the population of lactic acid bacteria in the intestine. Results showed that (1) dietary thyme and oregano additions are recommended to improve performance, (2) all aromatic herbs are recommended for villus development in the ileum, and (3) oregano, mint, and sage suppressed malondialdehyde formation.

## 1. Introduction

After the ban on the use of antibiotics as growth enhancers due to their harmful effects on humans through poultry products, researchers have, over the last 20 years, investigated the leaves and essential oils (phytobiotics) of medicinal aromatic plants in animal nutrition. Many medicinal aromatic plants from the Lamiaceae family are being explored as phytobiotic feed additives. Among these, thyme (*Thymus vulgaris*), sage (*Salvia officinalis*), mint (*Mentha piperita*) and oregano (*Origanum vulgare*) are the most commonly used [1,2].

Thyme is a medicinal aromatic plant of the Lamiaceae family with strong antioxidant [3], antimicrobial, and anti-inflammatory [3,4] properties. It has been reported that the antioxidant effect of *Thymus vulgaris* is due to the presence of flavonoids, with nearly 1% quercetin equivalents, contributing approximately 70% of its antioxidant activity [5]. In addition to its antioxidant effects, several studies have reported that thyme can act as a growth enhancer in broilers. For instance, the addition of 5 g/kg thyme powder (TP) [6,7,8], 0.6 g/kg TP [9], and 30 g/kg TP [10] to broiler feed improved growth performance.

Oregano (*Origanum vulgare*) is another Lamiaceae plant. Its leaves contain 1.88–3.5% essential oil, with carvacrol being the main component. Other compounds such as cineol, borneol, linalool, and γ-terpinene also contribute to its antimicrobial and antioxidant activity [11]. Ampode and Mendoza [12] reported that the inclusion of 5% oregano leaf powder in broiler feed improved performance. Similarly, Jahja et al. [13] found that water extracts of *Origanum vulgare* enhanced broiler performance. Roofchaee et al. [14] also reported that oregano essential oil improved live weight gain and feed utilization. Furthermore, Bauer et al. [15] noted that supplementing 5 g/kg of oregano powder to broiler diets significantly increased live weight gain. Ivanov and Bozakova [16] concluded that *Origanum vulgare* extract and its derivatives could be used to alleviate stress, oxidative stress, and improve intestinal microflora in both broilers and laying hens.

Mint (*Mentha piperita*) is another Lamiaceae plant, and its active compound, menthol, possesses antioxidant and anti-inflammatory properties, along with benefits for digestive health. Mint is also rich in antioxidants such as flavonoids, phenolic acids, and vitamin C. Asadi et al. [17] reported that the addition of 4.5 g/kg of mint powder to broiler feed improved performance. Mehri et al. [18] found that adding 10, 20, 30, or 40 g/kg of *Mentha piperita* powder to quail feed increased the population of lactic acid bacteria in the intestines and suppressed pathogenic bacteria, although it did not significantly affect performance. Similarly, Mirzadeh et al. [19] observed that a 0.5% mint extract in quail feed enhanced growth performance. Al-Kassie [20] also found improvements in growth performance in broilers fed 0.5% *Mentha piperita* powder.

Sage (*Salvia officinalis*), native to the Mediterranean region and also a member of the *Lamiaceae* family, is known for its antioxidant and antimicrobial effects [3]. It contains active compounds such as rosmarinic acid, caffeic acid, and flavonoids. Rosmarinic acid, in particular, is effective in scavenging free radicals and reducing inflammation. Farhadi et al. [21] reported that 0.5% sage powder and Alcicek et al. [22,23] reported that 5 g sage essential oil per kg of broiler diet enhanced growth performance.

Many studies support the potential effects of herbal powders on poultry performance. However, it is essential to include aromatic herbals at optimum levels. Higher doses may have adverse effects due to increased fiber content, which may hinder performance. This issue may explain why some studies have not observed any improvements in performance when higher doses of plant powders were used. As Tejeda and Kim [24] stated, the type and level of dietary fiber significantly affect intestinal development and feed digestion in poultry, which in turn affects performance. In addition, herbal powders are expensive products and their use at higher doses increases costs. In order for such products to be considered as feed additives, they must be added to the feed at a minimum level. In the literature review, although there are many studies using herbal powders individually, no studies were found to determine the effects of four different herbal powders at low doses on performance, intestinal histology, ileum mucin 2 gene expression, meat quality, and antioxidant effects in meat in quails. Therefore, the aim of this study was to determine the effects of low doses of thyme (*Thymus vulgaris*), sage (*Salvia officinalis*), mint (*Mentha piperita*) and oregano (*Origanum vulgare*) powder supplementation to quail diets (5 g/kg) on growth performance, internal organ development, cecum microbiota, intestine histomorphology, ileum mucin 2 gene expression, breast meat color properties and MDA formation.

## 2. Materials and Methods

### 2.1. Medicinal Aromatic Plants

Medicinal aromatic plants used in the study were thyme (*Thymus vulgaris*), sage (*Salvia officinalis*), mint (*Mentha piperita*) and oregano (*Origanum vulgare* L.), grown in the Medicinal and Aromatic Plants Application and Research Garden of Kırşehir Ahi Evran University in 2023, harvested in August and dried. Essential oils from aromatic plants were extracted using a microwave extraction system and HPLC analyses conducted at the university’s central research laboratory determined that thyme contains thymol 48.23%, oregano carvacrol 38.25%, mint menthol 40.8%, and sage camphor 33.51%. Vegetative parts of these plants were milled for 1 min in a blender and turned into powder and a homogeneous feed mixture was obtained by mixing the feed additives using a low-capacity feed mixer.

### 2.2. Animal Diet and Management

In this study, a total of 400 healthy quail chicks (1-day-olds) were purchased from a local hatchery in Kırşehir province. Quail chicks were distributed to 5 dietary treatments with 4 replicates (20 chicks). The treatment groups were as follows: (*C*) basal diet as control, (*T*) basal diet + 5 g/kg thyme, (*S*) basal diet + 5 g/kg sage, (*M*) basal diet + 5 g/kg mint, and *(O*) basal diet + 5 g/kg oregano. Chicks were reared on wood shavings on the ground throughout the experiment. Each cage (70 cm × 100 cm × 50 cm) was equipped with one feeder and a 2 L drinker. Feed and water were offered ad libitum, and a 24 h lighting program was applied throughout the experiment. Room temperature was set at 32 °C for 1 week and then reduced by 2 °C every week. The experiment was continued for 35 days. No vaccination was administered to chicks. During the study, starter diet (22.39% CP and 3080 kcal/kg ME) and grower diet (21.40% CP and 2850 kcal/kg ME) were purchased from a commercial feed company in powder form at Kayseri in Türkiye (Table 1).

Live weight and feed intake were recorded weekly. During the experiment, one death occurred in each of the control, thyme, and mint groups. At the end of the trial, 2 chicks (1 male and 1 female) close to the group average weight were slaughtered from each pen, and 8 chicks from each treatment, for 40 chicks in total, and their inner organ weights were weighed and values were standardized per 100 g live weight. Meat samples from chicks, in closed plastic bags to determine MDA formation and meat properties. The lightness (L*), redness (a*), and yellowness (b*) of breast and thigh meat from a total of 40 chicks, 2 from each replicate and 8 from each group, were measured using a Minolta Chrometer (CR-410; Konica Minolta, Osaka, Japan) in 3 replicates. The pH values of the breast and thigh meat were then measured with a pH meter (TESTO 205 pH meter (TESTO, Hampshire, UK). At the end of the slaughtering process, an average of 20 g of breast meat from each slaughtered quail was placed in sealed bags, sealed, and stored in the refrigerator for MDA analysis. MDA analyses were performed after the breast meats were stored in the refrigerator at 4 °C for 3 days.

### 2.3. Determination of the TBARS Value in the Breast Meat of Quail Chicks

Lipid oxidation levels in the samples were determined using the 2-thiobarbituric acid method described by Yesilbag et al. [25].

### 2.4. Intestinal Histomorphology

At slaughter, gut samples of each chick were taken from the jejunum and ileum, and stored in 10% formaldehyde. To illustrate the intestine histologically, the tissues were, firstly, dehydrated with a series of alcohols and embedded in paraffin blocks, then cut with a microtome at 5-micron thickness, and stained with a standard hematoxylin and eosin staining, then photographed with an AxioCam ERc 5s 5 megapixel digital microscope camera (Primo Star; Carl Zeiss Microscopy GmbH, Oberkochen, Germany) and measured using the ZEN 2012 SP2 image processing and analysis software.

### 2.5. Cecum Microbiota

After slaughtering, the cecum contents were immediately transferred to Petri dishes and taken to the microbiology laboratory. Here, 1 g of cecum contents was added to 9 mL of previously autoclaved distilled water and homogenized with a vortex. The resulting homogenate was diluted to a ratio of 108 and stock material was obtained, and the inoculation process was completed by inoculating the stock material on the necessary agars. For lactic acid bacteria, De Man–Rogosa–Sharpe agar (MRS agar (Merck 110660, Darmstadt, Germany), 3 days at 37 °C) was used. For *E. coli*, Eosin Methylene Blue agar (EMB agar (Merck 101347, Darmstadt, Germany), 2 days at 37 °C) was used. The counts of microorganisms were illustrated logarithmically (log_10_ CFU).

### 2.6. mRNA Expression

Primer sequences are presented in Table 2. Muc-2 gene expression was performed in the central research laboratory at the Kırşehir Ahi Evran University. Intestinal tissue (ileum section) of 3 individuals from each group to determine mucin 2 gene expression in quail was sampled. Ileum samples were transferred to cryo tubes (1.5 mL) with RNA Later Solution, and kept at −80 °C. Total mRNA was isolated using Qiagen RNeasy Kit (cat. no. 74804) and following the manufacturer’s instructions. The quantity of extracted RNA was measured at absorbance of 260–280 nm in a spectrophotometer Nanodrop-Multiskan go (Thermo scientific). RNA amount was calculated using the 1/RNA concentration equality. RNA samples were kept at −80 °C prior to cDNA synthesis. In the PCR plate, cDNA was synthesized by adding reactions consisting of oligo DT(20) (50 µm), RNaseOut, 5 First-strand Buffer, 0.1 M DTT, superscript III, 10mM dNTP, RNA and ddH_2_O. The 20 mL cDNA samples were measured in a Nanodrop Spectrophotometer and stored at −20 °C prior to qPCR. Real-time quantitative PCR processes were performed in Rotor-gene Q (Qiagen, Hilden, Germany). For this purpose, 1/1, 1/10, 1/100 and 1/1000 concentrations were prepared and measured in a Nanodrop Spectrophotometer. Subsequently, gene expression of the relevant gene was determined quantitatively by preparing 20 μL reactions containing 10 μL QiagenTag PCR master mix, 6 μL ddH_2_O, 1 μL cDNA, 1 μL forward primer, 1 μL reverse primer and 1 μL SYBR Green qPCR Master Mix (GoldBio, St. Louis, MO). The primer sequences for Mucin-2 and β-Actin were verified from Emadinia et al. [26]. Relative quantification of mRNA levels normalized with β-actin was calculated using the formula E = 10 (−1/slope) [27].

### 2.7. Statistical Analysis

All data were analyzed using one-way ANOVA with Duncan’s multiple comparisons using SPSS statistical software (IBM SPSS 25.0 for Windows; SPSS Inc., Chicago, IL, USA). The pen was used as the experimental unit for growth performance data, and the individual bird was used for the other parameters. Significance was set at *p*  <  0.05 and *p*  <  0.01. The results are presented as means and standard error of means (SEM). The statistical model was

Yij = µ + Ti + eij(1)

where Yij is the individual observation, µ is the overall mean, Ti is the effect of dietary treatments, and eij is experimental error.

## 3. Results

### 3.1. Performance Parameters

Performance parameters are given in Table 3. Body weight gain (BWG) was significantly affected by dietary treatments (*p* < 0.01). Quails supplemented with thyme (*T*) (173.94 g) and oregano (*O*) (173.78 g) showed significantly higher BWG compared to the control (*C*) (166.34 g), sage (*S*) (165.80 g), and mint (*M*) (164.39 g) groups. Feed intake (FI, *p* > 0.95) and feed conversion ratio (FCR, *p* > 0.09) were not affected by any herbal powders.

### 3.2. Internal Organs

The effects of dietary herbal powder supplementation in quail diets on internal organs are presented in Table 4, showing that dietary supplementation of herbal powders had no effect on internal organ weights and gut length.

### 3.3. Jejunum and Ileum Histomorphology

Jejunum and ileum histomorphological parameters are given in Table 5. The villi length (VL) in the jejunum was significantly influenced by dietary treatments (*p* < 0.001). The highest VL values were observed in quails supplemented with sage (*S*) (643.45 µm) and oregano (*O*) (643.63 µm), which were significantly greater than the control *T*_0_ (533.67 µm), thyme (*T*) (585.33 µm), and mint (M) (543.56 µm) groups. No significant differences were found in crypt depth (CD) among the groups (*p* = 0.354). The villi height to crypt depth ratio (VL/CD) showed a tendency toward significance (*p* = 0.082), with the highest ratio seen in the thyme (*T*) group (18.68).

In the ileum, significant differences were observed in all measured parameters too. Villi length was highest in the thyme (*T*) group (605.64 µm), followed by oregano (*O*) (539.53 µm), mint (*M*) (537.03 µm), and sage (*S*) (515.70 µm), all of which were significantly longer than in the control (C) group (433.58 µm) (*p* < 0.001). Crypt depth was also significantly affected (*p* < 0.001), with the deepest crypts in the sage (*S*) group (60.65 µm) and the lowest in the control group (39.02 µm). The VL/CD ratio differed significantly among groups (*p* < 0.001), with oregano (11.88) and thyme (11.43) showing the highest ratios, indicating improved intestinal morphology.

### 3.4. Breast Meat Color Properties and MDA Formation

The effects of dietary supplementation with thyme, sage, mint, and oregano powder on meat color, pH, and lipid oxidation (MDA levels) in quails are presented in Table 6. There were no statistically significant differences among the treatment groups in terms of breast meat color parameters L* (lightness, *p* = 0.35), a* (redness, *p* = 0.33), and b* (yellowness, *p* = 0.36). Similarly, the pH values of breast meat were not significantly affected by the dietary treatments (*p* = 0.72).

However, MDA levels, which indicate lipid oxidation, were significantly influenced by the dietary treatments (*p* = 0.05). The control group (*C*) showed the highest MDA value (1.32 mg/kg), while the lowest MDA level was observed in the mint group (*M*) at 0.54 mg/kg. MDA levels in the sage (*S*) and oregano (*O*) groups were also significantly lower than the control. The thyme group (*T*) showed a numerically lower MDA value (0.86 mg/kg) than the control, but this difference was not statistically significant from either the control or the other treatment groups. These results suggest that supplementation with sage, mint, and oregano powder at 5 g/kg can effectively reduce lipid oxidation in quail breast meat, thereby potentially improving meat quality during refrigerated storage.

### 3.5. Cecum Microbiota

The effects of dietary supplementation with thyme, sage, mint, and oregano on cecal lactic acid bacteria (LAB) and *E. coli* counts in quails are shown in Table 7. LAB counts were significantly affected by the dietary treatments (*p* = 0.03). The thyme (*T*), mint (*M*), and oregano (*O*) groups showed significantly higher LAB counts compared to the control (*C*) and sage (*S*) groups. The highest LAB count was recorded in the thyme group (7.15 log CFU/g), followed closely by oregano (7.03 log CFU/g) and mint (6.96 log CFU/g), while the lowest values were observed in the control (6.03 log CFU/g) and sage (5.98 log CFU/g) groups.

Although *E. coli* counts showed numerical differences among the treatment groups, these differences were not statistically significant (*p* = 0.07). The highest *E. coli* count was observed in the control group (7.28 log CFU/g), and the lowest was in the oregano group (6.83 log CFU/g).

These findings indicate that thyme, mint, and oregano supplementation can enhance beneficial gut microbiota by increasing LAB colonization, while potentially suppressing *E. coli* levels in the cecum.

### 3.6. Mucin Gene Expression

**Figure 1:**
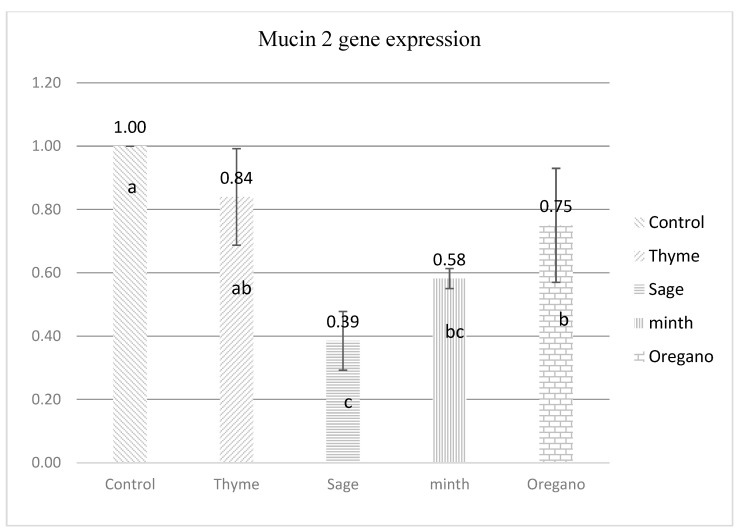
Differentiation of mucin 2 gene expression of quails grown with dietary supplementation of herbal powders (5 g/kg). Values represent means of four replicate pens with 3 birds from each treatment. There is a statistical difference between the averages shown with different letters a,b,c (*p* < 0.05).

Mucin gene expression of quails grown with dietary supplementation of herbal powders is presented in Figure 1. Dietary supplementation of sage, mint and oregano decreased ileum mucin 2 gene expression compared to that of the control group (*p* < 0.05). In particular, 5 g/kg sage supplementation suppressed gene expression the most. Thyme supplementation did not affect ileum mucin 2 gene expression compared to the control group (*p* > 0.05).

## 4. Discussion

The results of this study demonstrated that supplementing quail diets with 5 g/kg thyme and oregano vegetative powder significantly improved growth performance compared to the control, sage, and mint groups. In contrast, the inclusion of sage and mint vegetative powder did not yield any significant effect on performance parameters. When comparing these findings with previous studies, similar positive effects of thyme supplementation were reported. In broiler chickens, Deeb et al. [28] used 2–3 g/kg, Hassan and Awad [8] used 5 g/kg, and Belali et al. [29] used 150 mg/kg of thyme, all reporting increased live weight gain. Similarly, Soliman et al. [30] found that 5 g/kg thyme powder improved performance in quails. Regarding oregano, Alimohammadi et al. [31] (3 g/kg), Ampode and Mendoza [12] (30–50 g/kg), Ampode [32] (3%), and Giannenas et al. [33] (5–10 g/kg) all observed improved performance in broiler chickens. These findings align with the current study, where 5 g/kg thyme and oregano supplementation improved growth performance in quails. Although sage and mint were hypothesized to have positive effects on growth performance, no significant improvements were observed. This is consistent with the findings of Ocak et al. [34], who reported that 0.2% *Mentha piperita* leaf powder had no effect on growth performance in broilers. Similarly, Bulbul et al. [35] found that 200 mg/kg sage essential oil did not influence performance in broiler quails. However, contradictory findings have also been reported. Ocak et al. [34] found that 0.2% *Thymus vulgaris* and *Mentha piperita* leaf powder did not affect broiler performance, and Khatun et al. [36] reported no effect with 5 g/kg *Thymus vulgaris* and *Origanum vulgare*. On the other hand, Bonos et al. [37] noted that a sage-containing essential oil mixture enhanced broiler performance, while Abdel-Wareth et al. [38] found that 5–15 kg/kg mint powder improved performance in broilers. These inconsistencies across studies may be attributed to factors such as the timing of harvest and the duration between harvesting and use. In the current study, the plants were harvested in August 2023 and used fresh in trials conducted in September. This freshness may have contributed to the positive results observed with thyme and oregano, and also improved overall gut health by suppressing *E. coli* and increasing LAB colonization thanks to the thymol and carvacrol they contain, which in turn increased intestinal histological parameters and ensured better digestion. These positive effects also contributed to the improvement of quail’s performance. This is because thymol is a very strong antimicrobial product and has a very strong inhibitory effect on pathogens [39,40].

Therefore, further research is warranted to evaluate how storage duration affects the efficacy and phytochemical composition of such botanicals. Regarding internal organ development, the inclusion of low-dose phytobiotics had no notable effects. This finding is consistent with previous studies: Abdel-Wareth et al. [38] found no effect of mint on broiler internal organs, Vlaicu et al. [41] reported similar findings with thyme and sage, and Abd El-Aziz et al. [42] observed no impact of oregano on rabbits.

The supplementation of low doses (5 g/kg) of thyme, oregano, sage, and mint to quail diets had no significant effect on the color or pH values of breast meat. However, MDA analysis conducted three days post-slaughter revealed that these powders significantly enhanced the oxidative stability of the meat by suppressing MDA levels compared to the control group, indicating a notable antioxidant effect. Among the treatments, mint supplementation exhibited the greatest suppression of MDA. These findings are consistent with Masouri et al. [43], who reported that mint essential oil significantly reduced MDA levels in quail meat. Similarly, Deeb et al. [28] demonstrated that thyme leaf powder supplementation in broiler diets significantly suppressed serum MDA levels and enhanced superoxide dismutase (SOD) enzyme activity. Coskun et al. [44] reported that thyme powder supplementation suppressed MDA formation in quail meats. Also, Büyükkılıç Beyzi et al. [45] reported that thyme essential oil suppressed MDA in eggs. Saleh et al. [46] also found that supplementing laying hen diets with 0.5 kg/ton of sage and oregano powder significantly reduced MDA concentrations in liver tissue. In broilers, Bonos et al. [37] reported that a sage oil blend improved resistance of thigh and breast meat to lipid peroxidation without altering meat quality parameters. Vlaicu et al. [41] showed that 0.05% (500 ppm) thyme and sage essential oils increased antioxidant capacity and total polyphenol content in broiler meat. The findings of the current study are in agreement with the literature and suggest that low-dose supplementation of thyme, oregano, sage, and mint exerts a protective antioxidant effect on quail breast meat. These powders effectively will enhance shelf life by increasing resistance to lipid peroxidation, without negatively impacting meat quality traits such as color or pH.

Dietary supplementation of low doses of thyme, oregano, and mint powder to quail diets increased the colonization of lactic acid bacteria in the cecum, and although there was no statistically significant difference, all plants suppressed *E. coli* numerically, indicating that these plants directly contribute to the regulation of the intestinal microflora. Previous studies have reported that the essential oils contained in aromatic plants exhibit antimicrobial properties and suppress pathogenic bacteria. For example, Thyme [2,5,41,47,48,49], Oregano [14,37], Mint [18,50], and Sage [2,37,41,49] plants have been reported to suppress *E. coli* by exhibiting antimicrobial effects. Similarly, thyme [51], oregano [31], mint [18], and sage [51] plants have been reported to increase the LAB population. These effects are seen to be due to the antimicrobial effects of thymol [39] from thyme, carvacrol [52] from oregano, menthol [53] from mint, and camphor [54] from sage, which suppress the multiplication of *E. coli*. Therefore, the results obtained from this study are consistent with the results of previous studies. This study demonstrates that adding low doses of thyme, oregano, sage, and mint to quail diets suppressed *E. coli* in the quail’s ceca and increased lactic acid bacteria colonization, thus improving intestinal health.

The results of this study showed that the supplementation of low doses (0.5%) of sage and oregano powder to quail diets significantly increased villi length in the jejunum compared to the control, thyme, and mint groups. However, neither crypt depth nor the villi height-to-crypt depth (VH/CD) ratio was affected. In the ileum, both villi length and crypt depth increased across all treatment groups relative to the control. Notably, the VH/CD ratio was lowest in the sage-supplemented group, which appears to be attributable to the greater crypt depth observed in this group. These findings are largely consistent with previous studies. El-Sayed et al. [55] reported that *Origanum vulgare* essential oil supplementation in broiler chickens increased jejunal villi length without affecting the VH/CD ratio. In contrast, Xian et al. [56] observed that oregano essential oil increased both villi length and the VH/CD ratio. Farhadi et al. [21] found that supplementation with 0.2–1.2% sage powder enhanced ileum villi length in broilers. Similarly, Gümüş et al. [57] showed that thyme and rosemary essential oils increased intestinal villi length in broiler chickens, and Dehghani et al. [58] reported that various doses of mint essential oil improved intestinal villi length in quails.

Histomorphologically, the higher villi length in the groups supplemented with plant powders in the intestines of quails is the most obvious indicator of improved intestinal health. The main reason why plant powders increase villi length is that the thymol, carvacrol, menthol and camphor they contain exhibit antimicrobial properties, suppressing pathogens in the intestine, and as a result of this suppression, their destructive effects on the villi in the intestine are eliminated, allowing the villi to develop further. Abdelhamid et al. [58] reported that it caused damage to the intestinal mucosa in chickens infected with *E. coli*. Possible reasons for *E. coli* causing damage to the intestinal mucosa are: (a) by adhering to epithelial cells (adhesion) and causing disruptions in the functions of the cells, (b) by producing toxins causing cell death and disruption of tissue integrity (leaky gut), spread of pathogens and toxins in the body, inflammation and malabsorption, and (c) they cause various diseases (Colibacillosis, diarrhea, loss of appetite, weakness, feather ruff, etc.) [59]. It has been determined that the addition of plant powder to the feed of quails reduces the pathogenicity of pathogenic microorganisms in the intestines, suppresses their multiplication, reduces tissue damage in the intestines and increases the development of villi.

Thus, the results of the present study align with previous literature, suggesting that dietary supplementation with thyme, oregano, sage, and mint, even at low doses, generally exerts beneficial effects on intestinal morphology. Specifically, increased villi length indicates an expansion of the absorptive surface area in the small intestine, which may enhance nutrient absorption and overall digestive efficiency.

In this study, it was observed that low-dose supplementation of oregano, sage, and mint to quail diets resulted in a reduction in Muc-2 gene expression in the ileum, while thyme supplementation maintained expression levels similar to the control group. These findings were unexpected, as herbal powder supplementation was hypothesized to enhance Muc-2 expression due to its known role in supporting intestinal health. Muc-2 is a key gene responsible for the production of mucin, a major component of the intestinal mucus layer that plays a critical role in gut barrier function and host defense. Based on its importance, the expectation was that herbal powder supplementation would either maintain or increase Muc-2 expression. Previous studies support this assumption. For instance, Li et al. [60] reported that dietary supplementation with a eutectic mixture of thymol and carvacrol significantly increased Muc-2 expression in the ileum of broiler chickens. Mahmoud et al. [61] showed that oregano oil supplementation to quail diet increased Muc-2 gene expression. Similarly, İpçak et al. [62] found that encapsulated fennel seed (*Foeniculum vulgare* Mill.) essential oil supplementation significantly upregulated Muc-2 expression in all treatment groups compared to the control. However, contrasting results were reported by Duangnumsawang et al. [63], who found no effect of herbal powder supplementation on Muc-2 expression in broilers. In our study, the reason why Muc-2 gene expression did not change in the thyme group or decreased in other groups may be due to the application of herbal powder. This is consistent with the previous literature showing that herbal extracts or essential oils increase gene expression, while herbal powder application does not affect expression. However, these results need to be confirmed. Despite histomorphological and microbiological findings in this study suggesting that phytobiotics improved intestinal health such as increased villi length and enhanced microbial balance the unexpected decrease in Muc-2 expression (except in the thyme group) calls for further investigation. The inconsistency between gene expression and morphological outcomes indicates that additional regulatory mechanisms may be involved, or that other mucin-related or immune genes could be playing a compensatory role.

## 5. Conclusions

Dietary thyme and oregano powders can be used to increase the growth of quails. Thyme, oregano, sage, and mint powders can be used to suppress malondialdehyde in quail meat, decrease the formation of pathogenic bacteria in the intestines, and increase the lactic acid bacteria population; they can also be used to improve intestinal digestion and enhance villi morphology.

## Figures and Tables

**Table 1:** Composition of experimental diet (air-dry basis).

Ingredients	1–21 Days	22–35 Days
Maize, %	45.00	32.00
Soybean meal (44% CP), %	40.00	27.60
Meat-bone meal, %	3.00	4.40
Wheat bran, %	-	20.00
Sunflower meal, %	-	5.00
Soybean oil, %	6.50	5.20
Dicalciumphosphate, %	2.50	2.40
L-lysine HCl, %	1.00	1.20
DL-methionine, %	1.10	1.10
Salt, %	0.40	0.50
Vitamin premix, ^1^ %	0.25	0.30
Mineral premix, ^2^ %	0.25	0.30
Total	100	100
Nutritional contents		
ME, kcal/kg	3080	2850
Crude protein, %	22.39	21.40
Crude fiber, %	2.80	6.40
Ether extract, %	8.50	7.25
Calcium, %	1.049	1.00
Available phosphorus, %	0.380	0.48

^1^ Vitamin concentration supplied per serving of diet: vitamin A, 12,000 IU; vitamin D_3_, 2400 IU; vitamin E, 30 mg; 3 mg; niacin, 25 mg; vitamin B_6_, vitamin B_1_, vitamin B_2_, 7 mg; 5 mg; vitamin B_12_, 15 µg; and vitamin K3, 4 mg. ^2^ Trace mineral concentration supplied per calorie: pantothenic acid, 10 mg; folic acid, 1 mg; colin, 125,000 mg; biotin, 45 mg; Zn, 60 mg; Mn, 80 mg; Se, 150 µg; Fe, 80 mg; Cu, 5 mg.

**Table 2:** Primer sequences for mucin 2 gene in quails.

Genes	Forward (5′-3′)	Reverse (5′-3′)
Mucin-2	CCACAAGTCCTCCAGTACCTACA	AGGTTTCATAGTCACCACCATCTTC
ß-actin	CTGGCACCTAGCACAATGAA	CTGGTTGCTGATCCACATCT

**Table 3:** Performance parameters of quails with dietary supplementation of herbal powders (5 g/kg diet) ^1^.

Treatments	Control (*C*)	Thyme (*T*)	Sage (*S*)	Mint (*M*)	Oregano (*O*)	SEM	*p*-Value
IBW (g)	9.29	9.37	9.18	9.16	9.14	0.05	0.52
BWG (g)	166.34 ^b^	173.94 ^a^	165.80 ^b^	164.39 ^b^	173.78 ^a^	1.22	0.01
FI (g)	466.44	463.19	464.22	468.79	473.91	4.26	0.95
FCR	2.80	2.66	2.80	2.85	2.73	0.02	0.09

^1^ Values represent means of four replicate pens with 80 birds from each treatment. ^a,b^ There is a statistical difference between the averages shown with different letters (*p* < 0.05). SEM: standard error of means. IBW: initial body weight. BWG: body weight gain. FI: feed intake. FCR: feed conversion ratio.

**Table 4:** Internal organs of growing quails supplemented with (5 g/kg) dietary herbal powders (cm gr/100 gr BW) ^1^.

Treatments	Control (*C*)	Thyme (*T*)	Sage (*S*)	Mint (*M*)	Oregano (*O*)	SEM	*p*-Value
Heart	1.00	1.01	1.00	0.98	1.00	0.03	0.99
Liver	1.97	2.08	2.14	2.09	2.02	0.07	0.96
Gizzard	2.41	2.38	2.50	2.51	2.72	0.06	0.44
GL	32.10	34.10	33.59	37.21	32.30	0.73	0.17
EIO	5.38	5.47	5.64	5.58	5.73	0.49	0.79

^1^ Values represent means of four replicate pens with 8 birds from each treatment. SEM: standard error of means. GL: gut length. EIO: edible inner organs.

**Table 5:** Intestinal histology of growing quails with dietary supplementation of herbal powders (5g/kg) ^1^.

Treatments	Control (*C*)	Thyme (*T*)	Sage (*S*)	Mint (*M*)	Oregano (*O*)	SEM	*p*-Value
Jejunum							
VL (µm)	533.67 ^b^	585.33 ^b^	643.45 ^a^	543.56 ^b^	643.63 ^a^	9.77	0.001
CD (µm)	34.20	32.01	35.44	33.44	34.83	0.56	0.354
VL/CD	15.99	18.68	18.42	17.01	18.64	0.39	0.082
Ileum							
VL (µm)	433.58 ^c^	605.64 ^a^	515.70 ^b^	537.03 ^b^	539.53 ^b^	5.88	0.001
CD (µm)	39.02 ^d^	54.43 ^b^	60.65 ^a^	50.23 ^c^	46.98 ^c^	0.81	0.001
VL/CD	11.32 ^ab^	11.43 ^ab^	8.64 ^c^	10.87 ^b^	11.88 ^a^	0.17	0.001

^1^ Values represent means of four replicate pens with 8 birds from each treatment. ^a–d^ There is a statistical difference between the averages shown with different letters (*p* < 0.05). SEM: standard error of means. VL: villi length. CD: crypt depth.

**Table 6:** Breast meat color properties and MDA formation of quails grown with dietary supplementation of herbal powders (5 g/kg) ^1^.

Treatments	Control (*C*)	Thyme (*T*)	Sage (*S*)	Mint (*M*)	Oregano (*O*)	SEM	*p*-Value
L*	35.29	34.79	35.91	33.64	34.14	0.38	0.35
a*	10.67	10.46	11.51	10.73	10.83	0.16	0.33
b*	5.72	5.32	5.20	5.02	5.06	0.12	0.36
pH	6.10	6.19	6.17	6.04	6.14	0.04	0.72
MDA	1.32 ^a^	0.86 ^ab^	0.80 ^b^	0.54 ^b^	0.73 ^b^	0.08	0.05

^1^ Values represent means of four replicate pens with 8 birds from each treatment. ^a,b^ There is a statistical difference between the averages shown with different letters (*p* < 0.05). SEM: standard error of means. L*: lightness; 0 = black and 100 = white, a*: redness; −60 = green and 60 = red, b* yellowness; −60 blue and 60 = yellow.

**Table 7:** Cecum microbiota of quails grown with dietary supplementation of herbal powders (CFU/gr) ^1^.

Treatments	Control (*C*)	Thyme (*T*)	Sage (*S*)	Mint (*M*)	Oregano (*O*)	SEM	*p*-Value
LAB	6.03 ^b^	7.15 ^a^	5.98 ^b^	6.96 ^a^	7.03 ^a^	0.14	0.03
*E. coli*	7.28	6.85	6.94	7.02	6.83	0.05	0.07

^1^ Values represent means of four replicate pens with 8 birds from each treatment. ^a,b^ There is a statistical difference between the averages shown with different letters (*p* < 0.05). SEM: standard error of means. LAB: lactic acid bacteria. CFU: colony-forming unit.

## Data Availability

The original contributions presented in this study are included in the article. Further inquiries can be directed to the corresponding author.
